# Ancient Grain Flours with Different Degrees of Sifting: Advances in Knowledge of Nutritional, Technological, and Microbiological Aspects

**DOI:** 10.3390/foods12224096

**Published:** 2023-11-11

**Authors:** Tiziana Di Renzo, Giovanni Cascone, Giuseppina Crescente, Anna Reale, Valeria Menga, Maria D’Apolito, Stefania Nazzaro, Maria Grazia Volpe, Stefania Moccia

**Affiliations:** 1 National Research Council, Institute of Food Sciences, 83100 Avellino, Italy; tiziana.direnzo@isa.cnr.it (T.D.R.); giovanni.cascone@isa.cnr.it (G.C.); giuseppina.crescente@isa.cnr.it (G.C.); anna.reale@isa.cnr.it (A.R.); maria.dapolito@isa.cnr.it (M.D.); stefania.nazzaro@yahoo.it (S.N.); mgvolpe@isa.cnr.it (M.G.V.); 2 Council for Agricultural Research and Economics (CREA), Research Centre for Cereal and Industrial Crops, S.S. 673 m 25200, 71122 Foggia, Italy; valeria.menga@crea.gov.it

**Keywords:** Risciola, Carosella, Saragolla, ATR-FTIR, chemical composition, amylose–amylopectin ratio, glycemic index, microbiological quality, gluten content, farinographic properties

## Abstract

Ancient grains have gained considerable attention in recent years, as some research suggests they may be healthier than modern wheat. The present study aims to evaluate the chemical, rheological, and microbiological features of three Southern Italian cultivated ancient wheat varieties: Risciola, Carosella, and Saragolla. ATR-FTIR analyses were performed on the finely ground grain samples of the three varieties. The selected grains were ground with a stone mill, and different sifting degrees (whole—100%, type 1—80%, and type 0—72%) were evaluated. The flours showed a good nutritional profile, a higher amylose/amylopectin ratio, and a lower glycemic index than the literature. The gluten index of the samples was in the range 2.6–28.9%, and the flours can be classified as weak, having a value <30%. The farinographic test showed a short development time, low dough stability, a high softening degree, and water absorption, which increased with the degree of sifting. Microbiological analyses performed on flours from ancient grains at different degrees of sifting show their safety, according to their microbiological parameters, which fall within the legal microbiological requirements established by the European Commission Regulation (EC).

## 1. Introduction

Cereals are essential to a healthy and balanced diet since they provide nutrients and energy; they are an important source of complex carbohydrates, proteins, fiber (soluble and insoluble), B vitamins, vitamin E, as well as minerals (iron, zinc, magnesium, and selenium). Worldwide, wheat is the most cultivated cereal, and its products are widely consumed [1]. Its high yield and ability to adapt to a variety of climates make wheat an extremely versatile and stable crop [2]. World production of this crop is steadily expanding, hitting 779 million tonnes in the 2021–2022 campaign, and projections indicate that 781 million tonnes will be produced in 2023 [3]. Most of the produced wheat (95%) is *Triticum aestivum* (soft wheat), with *Triticum durum* making up the remaining 5% [4].

Several processes are involved in the manufacturing of wheat, with milling being the main one. When wheat is milled, the kernels are broken down and the endosperm is separated from the bran and germ [5]. The two predominant techniques for milling wheat are stone and roller milling. The process of stone milling uses multiple physical forces simultaneously to grind wheat between two stones, producing a flour that has all the kernel fractions. Depending on the goal, the process will end here for whole-wheat flour, while centrifugal sifters are used for refined white flour [6]. In roller milling, the endosperm is separated from the bran and germ, which is then sieved and ground further [7].

After milling, the bran is removed from the flour through sifting. Wheat flours are categorized into five types based on the degree of sifting: flour type 00, with a sifting of 50%; flour type 0, with a sifting of 72%; semi-wholemeal type 1, with a sifting of 80%; semi-wholemeal type 2, with a sifting of 85%; and wholemeal flour, with a sifting of 100% [8].

Based on their genetic heritage, cereals are classified as “modern” or “ancient” [9]. Despite the lack of a clear definition, it is widely acknowledged that ancient grains have not undergone any modern breeding or selection [10]. The term “ancient wheat” refers to three species: *Triticum monococcum*, a diploid wheat; *Triticum dicoccum*, a primitive tetraploid wheat called “spelt”; and *Triticum spelta*, a hexaploid grain called “spelta” [11]. These grains, cultivated and consumed since ancient times, have been gradually replaced by more productive and disease-resistant modern wheat varieties in what is commonly referred to as the “Green Revolution”. This term describes research and technology transfer that took place between the 1930s and the late 1960s [4]. The demographic growth in those years led to a sharp rise in food consumption, particularly in developing countries [2]. This phenomenon led to an increase in global cereal production and a radical shift in wheat-cultivation and selection methods, owing to two factors: firstly, the widespread availability of fertilizers, primarily nitrates and phosphates; secondly, agricultural production-related processes were becoming industrialized. As a result, wheat was selected based on the high gluten resistance, which was necessary for industrial processes and high crop yields [12]. Additionally, dwarfing genes for grains were introduced during the “Green Revolution” to increase wheat production [2]. Thus, this reduction in plant height, known as “dwarfing”, from around 150 to 180 cm to less than 50 cm, has emerged as the most obvious feature distinguishing the ancient wheat from the modern type [12].

Even though these programs have improved production and technology, they have also weakened wheat’s genetic variability and diminished its nutritional and nutraceutical properties [4].

Although, interest in ancient grains is growing exponentially as, despite having a lower yield than modern cultivars, they seem to have shown greater sustainability and better nutritional profiles [13].

In fact, ancient cultivars are naturally suitable for environmentally friendly organic cultivation systems, as opposed to modern wheat types, which require many agrochemical inputs like fertilizers, pesticides, and herbicides and are vulnerable to climate change and global warming [2,10].

Another aspect is that ancient grains have higher micronutrient levels than modern ones, which might be attributed to the shrinkage of the root system caused by the insertion of semi-dwarfing genes in modern grains, decreasing the plant’s ability to take up micronutrients from the soil [14].

In terms of protein content, it is higher in ancient cultivars than in modern ones; at the same time, the gluten content is also higher, but the quality is completely different with much weaker structure and strength [12]. A preliminary in vitro and in vivo study suggested that ancient grain varieties are better tolerated by people with non-celiac wheat sensitivity and irritable bowel syndrome, resulting in a longer diagnostic delay than modern grains [15].

As well, another study compared two ancient wheat species (spelt and einkorn) with a modern one (common wheat) for physicochemical composition and mineral elements, as well as performed SEM analysis of wheat grains’ microstructure. The ancient species showed higher values with regard to chemical composition (ash, protein, wet gluten and lipid content); moreover, einkorn’ SEM analysis revealed smaller starch-granule diameter and tighter protein bonds than the other two varieties, making it easier to digest [16].

Furthermore, the increased interest in ancient wheat species can also be attributed to the amount of phytochemicals, including polyphenols, carotenoids, and phytosterols, which are essential for preventing chronic diseases and degenerative conditions [17].

The present study aimed to evaluate the physicochemical, microbiological, and rheological properties of flours from three ancient wheat varieties grown in Southern Italy, two soft wheat (Risciola and Carosella), and one durum wheat (Saragolla), based on the degree of sifting (whole wheat, type 1 and 0). The ability of FTIR spectroscopy in ATR mode to discriminate between different cereal varieties was used on finely crushed whole cereals. At the same time, the amylose/amylopectin ratio and the glycemic index of flours derived from ancient grains were determined.

## 2. Materials and Methods

### 2.1. Chemicals

All chemicals were of analytical reagent grade. All microbiological media used were purchased from Oxoid (Milan, Italy), unless otherwise specified and were prepared according to the supplier’s instructions.

### 2.2. Raw Materials

**Figure 1:**
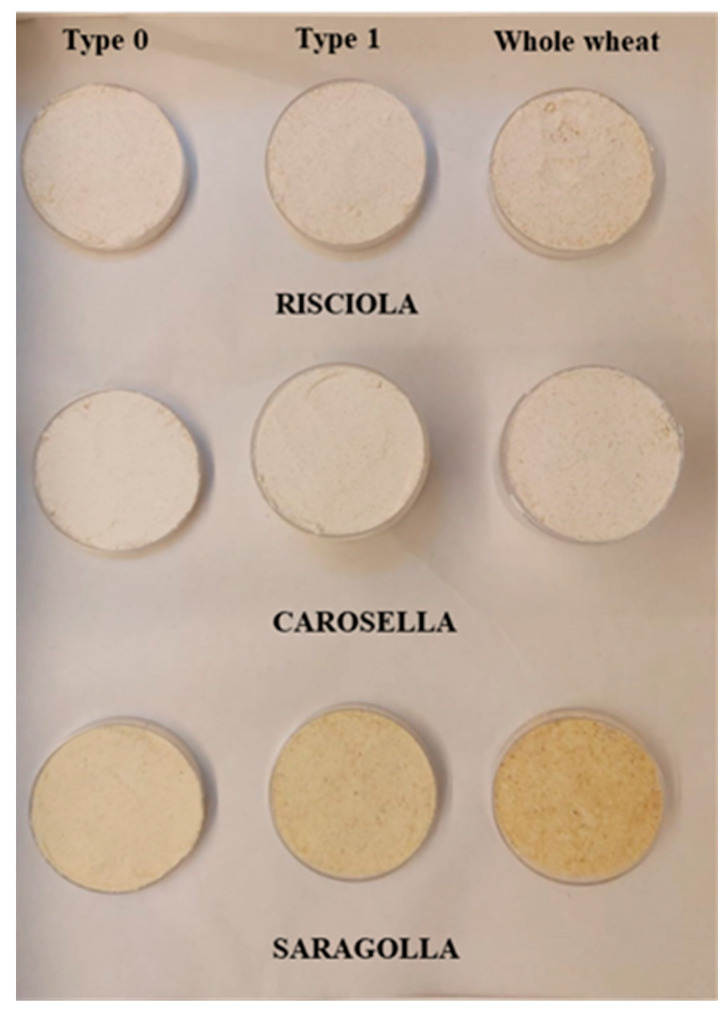
Shown from left to right are the different degrees of sifting (whole wheat, type 1, and type 0) of ancient grain flours (Risciola, Carosella and Saragolla) from Southern Italy.

Three varieties of ancient grains (Risciola, Carosella, and Saragolla) were supplied by the *Lucifero* farm in Zungoli (Avellino, Italy) in September 2022. Risciola and Carosella are soft wheat varieties, while Saragolla is durum wheat. The different grains were grown in the same area and growing season to minimize variations due to agronomic and environmental variables. To obtain flour, the grains of each variety were milled with a stone mill, carefully controlling the temperature throughout the milling process at the *Mulino Bencivenga* farm in Alvignano (Caserta, Italy), and at different sifting degrees: whole wheat (100% sifting degree), type 1 (80 % sifting degree), and type 0 (72% sifting degree, [18]). After grinding, flour samples were stored at 4 °C until analyses (Figure 1).

The samples under investigation will now be named: Risciola whole wheat (Rw); Risciola 1 (R1); Risciola 0 (R0); Carosella whole wheat (Cw); Carosella 1 (C1); Carosella 0 (C0); Saragolla whole wheat (Sw); Saragolla 1 (S1); and Saragolla 0 (S0).

### 2.3. ATR-FTIR Spectroscopy

The ATR-FTIR spectra of the three grinding types of cereal were recorded at resolutions of 8 cm^−1^ with 32 scans in the mid-IR region (4000–650 cm^−1^), using a Spectrum 400 spectrophotometer (PerkinElmer, Waltham, MA, USA). Briefly, 5 mg of the finely ground grain sample was placed directly on the surface of the germanium crystal, and the spectrum was recorded. The crystal surface was cleaned after each spectral collection using 0.1% (*w*/*v*) Alconox solution (Alconox Inc., New York, NY, USA). To test repeatability, analyses were performed in triplicate, and average spectra were used. The reference spectrum was measured against air. Spectra were elaborated using PE Spectrum software version 10.5.1, purchased with the instrument.

### 2.4. Color, pH and Water Activity Measurement

The color of ancient grain’s flours with different degrees of sifting was measured using CIE (Commission Internationale de l’Eclairage) L*, a* and b* color system, where L* describes brightness; a* is redness; and b* is yellowness. Color measurements were performed in triplicate with a colorimeter (model CR300, Minolta Italia S.p.A., Milan, Italy) as reported previously [19].

The pH was measured on 10 g flour samples after homogenization in 90 mL distilled water (H_2_O_d_) for 2 min in a Stomacher laboratory blender (BAG MIXER 400, Interscience, Saint-Nom-la-Bretèche, France) with a Medidor PH Basic 20 pHmeter (CRISON, Barcelona, Spain).

The water activity (a_w_) was determined at 20 °C using a calibrated a_w_ meter Rotronic (model HP23, HygroPalm, Bassersdorf, Switzerland) as described by Santagata et al., (2022) [20].

### 2.5. Proximate Composition Analysis

Moisture was assessed by drying 20 g of each sample at a temperature of 103 °C (±2 °C) in an isothermal oven until a constant weight was reached. The ash content was carried out through a gravimetric method which involves the incineration of an aliquot of the sample (5 g) at a temperature of 550 ± 10 °C in a muffle furnace until complete combustion of the organic substance and the achievement of a constant mass. Kjeldhal and Soxhlet methods were used to determine total protein and lipids, respectively [21].

The determination of the total fiber content (TDF), insoluble (IDF), and soluble (SDF), was carried out using an enzymatic and gravimetric method described by [22,23,24], using a kit provided by Megazyme International^©^ (Megazyme International, Wicklow, Ireland). The procedure was based on a sequential enzymatic incubation mimicking digestion using thermostable α-amylase, protease, and amyloglucosidase followed by an analysis of enzymatic digest.

Carbohydrate content was calculated by subtracting the mean values of other parameters from 100 [25]:

% carbohydrate (simple and complex) = 100 − (% moisture + % ash + % protein + % fat + % fiber)

### 2.6. Determination of the Amylose and Amylopectin Contents of Starch

For the analysis of amylose content and amylose/amylopectin ratio, a Megazyme International^©^ kit was used; it works by separating amylose and amylopectin, precipitating the latter with concanavalin-A, and then performing elimination using centrifugation [26]. The manufacturer’s instructions have been strictly followed. The absorbance was measured at 510 nm (DU730 UV-Vis Spectrophotometer, Beckman Coulter, Milan, Italy) to determine the sample’s amylose content.

### 2.7. In Vitro Starch Digestibility and Predicted Glycemic Index (pGI)

The in vitro digestion of the flours was conducted according to the method described elsewhere [27]. Briefly, 5 g of sample was suspended in 50 mL of H_2_O_d_ and 5 mL of maleate buffer (pH 6) and was then allowed to equilibrate at 37 °C for 15 min. After adding 100 µL of amyloglucosidase (A 7095, Sigma-Aldrich, Milan, Italy) and 1 mL of pancreatin (2 g/100 g; P7545, Sigma-Aldrich), this suspension was incubated for 180 min at 37 °C in a shaking water bath.

Aliquots of 500 µL were collected at 0, 20, 60, 120, and 180 min and incubated with 2 mL of EtOH for 1 h. Thereafter, the solution was centrifuged (Neya 16R; 2000× *g* for 2 min).

The supernatant (50 µL) was mixed with 250 µL of amyloglucosidase (EAMGDF; Megazyme International Ireland Ltd., Wicklow, Ireland; 1 mL/100 mL in 0.1 M sodium acetate buffer, pH 5.2) and incubated at 20 °C for 10 min. Then, to this, 750 µL of DNS solution (10 g/L 3,5-dinitrosalicylic acid, 16 g/L NaOH, 300 g/L Na-K tartrate) was added, mixed, and boiled for 15 min. The solution was cooled in cold water for 1 h, and 4 mL of water was added before measuring absorbance at 530 nm (DU730 Beckman Coulter, Brea, CA, USA).

#### Application of a Mathematical Model

The glycemic index (GI) was calculated from the starch digestion expressed as glucose concentration in the digestion solution at different times (0, 20, 60, 120, and 180 min).

The calibration for extrapolation of concentrations from absorbances was conducted with solutions at known glucose concentrations. According to the equation described by Goñi et al. (1997) [28], the kinetics of starch hydrolysis were calculated:

$$C=C_{\infty }\left(1-e^{-kt}\right)$$

where *C* is the concentration at time *t*; *t* is the digestion time; *C_∞_* is the equilibrium concentration of glucose (from hydrolyzed starch); and *k* is the kinetic constant. The parameters *C_∞_* and *k* were calculated by minimizing the root mean square between the experimental and theoretical values of the curve. The tool to obtain these values was the Excel solver which uses a non-linear GRG resolution method.

The area under the curve (*AUC*) was calculated by integrating the glucose-concentration curve.

$$\int_{C\left(t=0\right)=0}^{C(t)}C\;dC=AUC=\int_{0}^{t}C_{\infty }\left(1-e^{-kt}\right)\;dt$$

The following mathematical expression is the result of the integration of the first equation from 0 to 180 min, and it was used for calculating the *AUC* [29]:

$$AUC=C_{\infty }\left(t_{180}-t_{0}\right)-\frac{C_{\infty }}{k}\left[1-e^{-k\left(t_{180}-t_{0}\right)}\right]$$

where *t*_0_ = 0 min indicates the start of digestion, and *t*_180_ = 180 min is the maximum observation time of digestion.

The ratio of the *AUC* of each sample to the *AUC* of the control white bread was used to determine the hydrolysis index (*HI*):

$$HI=\frac{{AUC}_{sample}}{{AUC}_{white\;bread}}\cdot 100$$

The *HI* value obtained for each sample was used in the equation given below to determine the *pGI* [29]:

pGI=39.71+0.549·HI

### 2.8. Quality Traits Assessment

Gluten content (GC) and gluten index (GI) were determined by the Glutomatic System (Perten, Sweden) according to the AACC method 38-12 [21]. Data were expressed as grams per kilogram of dry matter (g kg^−1^ DM).

Farinographic properties of dough were determined according to the standard methods [30] using a Farinograph (BRABENDER OHG, Duisburg, Germany). The farinographic parameters such as water absorption (WA, %), developing time (DT, min), stability time (ST, min), and degree of softening (DS, FU) were recorded.

### 2.9. Microbiological Analysis

For microbiological analyses, 10 g of each flour sample was aseptically transferred into a sterile stomacher bag and diluted with 90 mL of physiological solution (9 g/L NaCl). After 1 min of shaking in a Stomacher apparatus (BAG MIXER 400, Interscience, France), the samples were serially diluted and plated. Total mesophilic bacteria were determined on Plate Count Agar after incubation at 28 °C for 48 h. *Enterobacteriaceae* were estimated on VRBGA after 36 h incubation at 37 °C. Total and faecal coliforms were counted on VRBA after 36 h incubation at 37 °C and 44 °C, respectively. Enterococci were counted on Slanetz and Bartley medium after 36 h incubation at 37 °C. Yeasts and molds were quantified on YPD agar plates, as reported by Reale et al., (2013) [31].

Lactic acid bacteria (LAB) were counted on De Man, Rogosa, and Sharpe (MRS) agar and 4 mg/100 mL cycloheximide (SIGMA Aldrich, Taufkirchen, Germany), after incubation at 28 °C for 72 h under anaerobic conditions (Gas Pack AnaeroGen TM, OXOID S.p.a., Milan, Italy). The determination of *B. cereus* was carried out using *Bacillus Cereus* Agar Base (PEMBA) after incubation for 24 h at 37 °C.

The presence of *Salmonella* spp. was investigated according to ISO standard 6579. Briefly, 25 g of each sample were suspended in 225 mL of Buffered Peptone Water and incubated for 18 h at 37 °C; 0.1 mL pre-enrichment was selectively enriched in 10 mL of Rappaport Vassiliadis Enrichment Broth and incubated for 24–48 h at 42 °C. Selective isolation was carried out on Salmonella Shigella Agar after incubation for 24 h at 37 °C.

The results of viable counts were expressed as a log of colony-forming units per gram of flour (CFU/g).

### 2.10. Statistical Analysis

For ATR-FTIR, statistical analysis was performed using the Spectrum AssureID software (Version 4.3, Perkin Elmer, Waltham, MA, USA), which uses the algorithm SIMCA (Soft Independent Modeling Class Algorithm), as a chemometric approach to separate models based on Principal Component Analysis (PCA).

The data were expressed as the means ± standard error of the mean of three independent measurements. The statistical significance was evaluated with a one-way analysis of variance (ANOVA) with Tukey’s HSD test. A *p*-value of <0.05 was considered statistically significant.

For proximate composition, amylose and amylopectin content and calculation of glycemic index, the significance between type 0 vs. type 1 and wholemeal flours was measured using the Student’s *t-test*. The significance level was fixed at 0.05 for all the statistical analyses; values with *p*-value < 0.05 were considered statistically significant.

## 3. Results and Discussion

### 3.1. ATR-FTIR Spectral Analysis

The ATR-FTIR analysis was performed on finely whole ground grains, and the FTIR data sets were analyzed using PCA to investigate which main chemical features were responsible for possible variations among the ancient grain samples.

**Figure 2:**
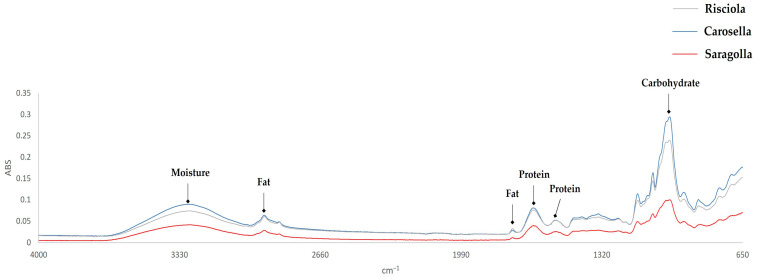
Overlapping infrared spectra of selected cereals, appropriately grounded: Risciola (grey line), Carosella (blue line), and Saragolla (red line).

The ATR-FTIR spectra of Risciola, Carosella, and Saragolla wheat samples are shown in Figure 2. The spectra clearly show the infrared bands characteristic of organic functional groups associated with proteins, carbohydrates, lipids, and moisture content. In both durum and soft wheat samples, the spectral bands were observed at ~ 995 cm^−^^1^ (carbohydrates); ~1645 and ~1545 cm^−^^1^ (proteins); ~1742 and ~2927 cm^−^^1^ (fats); and ~3288 cm^−^^1^ (moisture) [32].

**Figure 3:**
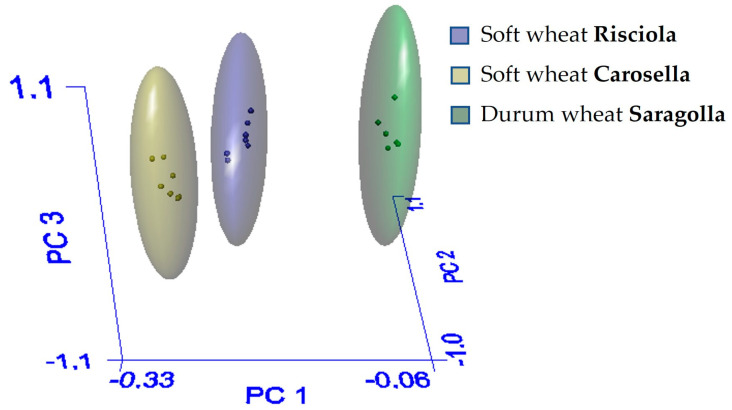
Three-dimensional PCA score of the three grinding cereals Risciola, Carosella, and Saragolla derived from SIMCA.

Durum and soft wheat samples show the same trend; nevertheless, the intensities of the ATR-FTIR bands of soft and durum wheat samples differed. Principal component analysis (PCA) was used to summarize the interrelationships between the physicochemical, nutritional, and structural properties of wheat samples based on their typology. As seen in Figure 3, the 3D-PCA scores plot generated with the Soft Independent Models of Class Analogy (SIMCA) method, produced three clusters.

The differences between the three samples were only significant in the Carosella/Saragolla combination, not in Risciola/Carosella, and, interestingly, in Risciola/Saragolla, indicating that the Risciola variety possesses qualitative traits of both soft and durum wheat. In fact, the Inter Material or Interclass Distance (IMD) is greater than three only for Carosella/Saragolla (Table 1, panel a): if the distance between two samples is less than three, the samples are not well separated and thus fall into the same class. Furthermore, the percentage of recognition and rejection in the optimal model must be as near to 100% as possible. A recognition number of 100% implies that all samples have been properly categorized, whereas a rejection rate of less than 100% shows that some samples have not been classified (Table 1, panel b) [33].

### 3.2. Flour’s Color, pH and Water Activity

Table 2 shows the results of color, pH, and water activity (a_w_) of ancient grain flours (Risciola, Carosella and Saragolla) with different degrees of sifting. Color values differed significantly (*p* < 0.05) depending on both variety and degree of sifting. In all varieties, there was an increase in L* values from “whole wheat” to “type 0” flour.

In particular, regardless of the degree of sifting, Carosella flour was lighter than Risciola and Saragolla with brightness values (L*) ranging between 70.51 ± 0.21 (Cw) and 71.29 ± 0.16 (C0). The lowest L* values were recorded for Saragolla flour samples, which registered values between 61.26 ± 0.15 (Sw) and 66.18 ± 0.12 (S0). Significant differences were also noted for color parameters a* and b*. In all cases, the whole-wheat-flour samples showed a greater redness (a*), and flours of the Saragolla variety showed the highest values of this parameter. In contrast, the lowest values were recorded for the Carosella variety. However, regarding the b* parameter, the durum wheat samples’ Saragolla showed more yellowness (b*) than Carosella and Risciola flours. This parameter showed for Saragolla flours values ranging between 15 ± 0.05 (S0) and 17.30 ± 0.28 (Sw). The lowest values of parameter b* were recorded for the Carosella variety (Table 2). The color of flours, which is related to the sifting rate and the genotypic characteristics of the grains, is a very important quality parameter, as it influences consumer choice and acceptance of finished products [34]. Generally, an intense yellow color characterizes durum wheat flours, while whiter ones are typical of common wheat flour [35]. Whole-wheat flours were darker (i.e., with lower L values) because they were obtained by milling whole wheat grains. These results are in agreement with those reported by Hidalgo et al. (2017) who found that in whole-wheat flours, color is affected by the presence of dark bran fractions [36]. As the degree of sifting decreased, the flours became lighter in color, since they were obtained by milling the core of the grain. The a* and b* values decreased as the sifting progressed, resulting in more green and less reddish and more blue and less yellow samples.

Table 2 also shows the pH and water-activity values of ancient grain flours. In particular, the pH values showed no significant differences among the flour samples analyzed, ranging between 6.05 ± 0.04 (Rw) and 6.13 ± 0.04 (S0). Our results agree with those reported by Cardoso et al. (2019) who found similar pH values in wheat flours from Portugal [37].

Although pH is an essential parameter that can influence food characteristics such as aroma, texture, and flavor, few studies have been conducted on determining the pH of wheat flours. In our study, pH values close to neutrality were recorded.

Ancient grain flours differ significantly also in a_w_ values, and Carosella variety flours showed the highest values (~0.512). Water activity and moisture content are key parameters in defining food stability. Our results showed low water-activity and moisture values were able to ensure good microbial stability of flours during storage, as reported by other authors [38,39].

### 3.3. Proximate Composition

The proximate composition of flour samples is summarized in Table 3.

The D.P.R. of 9 February 2001, n. 187 on the manufacture and marketing of flour establishes parameters such as moisture content and minimum protein levels, as well as variance restrictions. This regulation suggests that soft and durum wheat flours intended for sale may have a maximum relative humidity of up to 15.50 if the packaging specifies this amount [8]. The highest moisture-content value was found in C0 flour (12.71 ± 0.01) and the lowest in Rw flour (10.62 ± 0.07) (Table 3). These results are within the legal maximum values. A variety of factors affect the moisture content of flour, including the external temperature and humidity, agronomic practices adopted and grain conditioning during milling and storage.

Regarding the ash content, the whole-wheat-flour samples showed the highest values ranging from 1.20 ± 0.08 (Cw) to 2.04 ± 0.05 (Rw) (Table 3), according to the regulation [8]. This is owed to the presence of a high mineral concentration in these samples, which do not incinerate at 550 °C [37]. In fact, most of the minerals are concentrated in the outer layer of the wheat grain which is left in whole-grain flour [1].

The protein content is related to both genetic and non-genetic variables because nitrogen-rich soils may modify protein levels [40,41]. In Table 3, it is also visible that the percentage of proteins varied from 8.06 ± 0.01 (C0) to 10.54 ± 0.03 (R1) with 9.47 as the average. It was found that in Indian wheat cultivars, protein levels ranged from 8.65% to 12.02% and from 8.26% to 12.85% [42,43]. Moreover, in a comparison of commercial and monovarietal wheat flour from Peru, it was found that the average protein level was 9.64% and 8.02%, respectively [40].

Lipids are found throughout the wheat grain and mainly in the germ and aleurone, with a relatively smaller amount in the endosperm [44,45]. Lipid values (%) in our samples ranged between 1.00 ± 0.07 (C0) and 2.63 ± 0.01 (Sw). As expected, lipid content was higher in whole-grain flours than in refined ones (Table 3), as described by Ntuli et al. (2013) [46].

Fibers differ according to the sifting degree of flour: the higher the degree of sifting, the higher the fiber content (Table 3). Depending on their water solubility, dietary fibers can be distinguished into insoluble fiber (IDF) and soluble fiber (SDF) [47]. Soluble dietary fiber does not contain cellulosic polysaccharides, as is the case of insoluble fiber, which is composed primarily of cell-wall components like cellulose, hemicellulose, and lignin [25]. Generally, cereals contain high amounts of IDF, and whole grains are an excellent source [48,49]. While the germ includes vitamins, minerals, lipids, and some proteins, the bran is the primary source of phenolic acids, minerals, and dietary fiber. During the milling process to produce wholemeal flours, the external multilayers (bran) and germ are preserved along with the main starchy portion of the grain, as opposed to flours of different sifting degrees, where the various components are separated [50].

Complex carbohydrates constitute most of the wheat (61–65%) [51]; in detail, the carbohydrate content (%) of the flours investigated varied from 61.61 ± 0.10 (Rw) to 70.78 ± 0.07 (C0). Similar results in wheat samples from Greece were reported by Frakolaki et al. (2018) (67.78 g/100 g) [52]. Moreover, the carbohydrate content increases in more refined flours in accordance with Călinoiu and Vodnar (2018) [1].

### 3.4. Determination of the Amylose and Amylopectin Contents in Starch

Starch, the most important storage polysaccharide, and the most abundant constituent of many plants, is a substantial component of wheat grain, accounting for more than half of total endosperm dry matter [53]. The main components of starch are amylose (about 20%) and amylopectin (about 80%) [54]. Amylose is a linear polymer composed of glucopyranosyl units that are linked together by α-(1,4) glycosidic bonds; amylopectin, on the other hand, is a branching polymer composed of glucopyranosyl units connected by α-(1,6) links [55]. Different starches have different amylose/amylopectin ratios, which can influence the granular structure, physicochemical properties, and nutritional value of the flours and the resulting food products [53,56].

**Figure 4:**
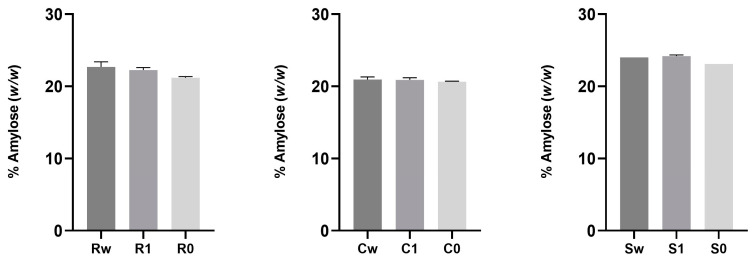
Percentage content of amylose (*w/w*) in flours obtained from ancient grains with different degrees of sifting (whole wheat, type 1, and type 0). Data are expressed as mean ± SD. The absence of symbols indicates non-significant differences between samples. Abbreviations: Rw: Risciola whole wheat; R1: Risciola 1; R0: Risciola 0; Cw: Carosella whole wheat; C1: Carosella 1; C0: Carosella 0; Sw: Saragolla whole wheat; S1: Saragolla 1; and S0: Saragolla 0.

Figure 4 displays the percentage content of amylose in the flours under investigation with different degrees of sifting.

The amylose content (*w*/*w*, %) of the starches in the flour samples evaluated in this study, ranged between 20.65 ± 0.03 (C0) and 24.22 ± 0.14 (S1), as shown in Figure 4. Amylose content in common wheat cultivars was determined to be on average 16.91% by Valková et al., (2021) [57]. In comparison to Murugadass and Dipnaik (2018) and Imanningsih (2012) [58,59], who reported values of amylose of 12.7% and 10.2% in wheat starch, respectively, the recorded data showed a greater amylose concentration. Furthermore, differences related to the type of wheat (soft and durum), are observed; according to Singh et al., (2012), the amylose content is on average higher in durum wheat than in soft wheat [60].

The amylose/amylopectin ratio was also assessed in this study; based on the data reported in Table 4, the amylose/amylopectin ratio does not differ between different sifting degrees. The main reason for this is that starch is mainly located in the endosperm, the innermost layer of the grain [47], which is not removed during the sifting process.

This ratio affects both gastric health and postprandial blood-glucose levels, as well as lipid metabolism through its influence on starch digestibility [57].

### 3.5. In Vitro Starch Digestibility and Predicted Glycemic Index (pGI)

In the classification of foods, the glycemic index is measured on a scale from 0 to 100; it is often classified into three categories: low (equal to or less than 55), medium (56–69), and high (above 70) [61].

**Figure 5:**
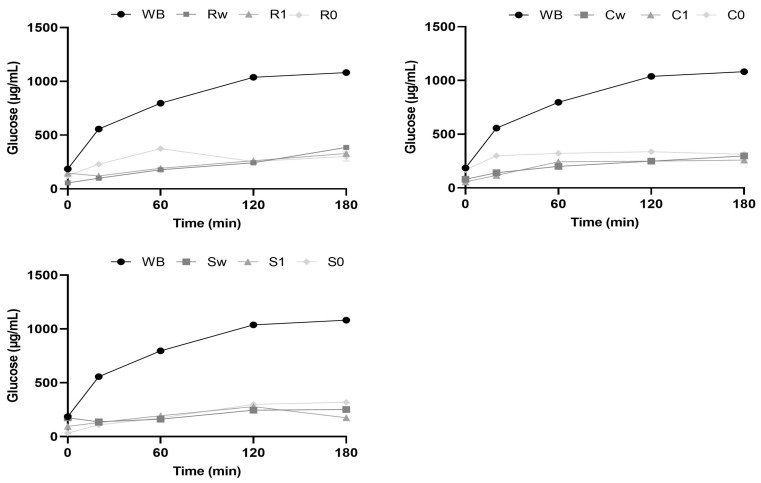
Kinetics of starch hydrolysis under in vitro conditions for the different samples analyzed. Data are expressed as mean ± SD of three independent experiments. Abbreviations: WB: white bread; Rw: Risciola whole wheat; R1: Risciola 1; R0: Risciola 0; Cw: Carosella whole wheat; C1: Carosella 1; C0: Carosella 0; Sw: Saragolla whole wheat; S1: Saragolla 1; and S0: Saragolla 0.

Through in vitro digestion kinetics, the pGI of the samples was assessed; the hydrolysis curves of the ancient grain flours, are illustrated in Figure 5, and the samples were compared to white bread (WB) as a reference. The results regarding the ancient grain flours showed lower released glucose than white bread. Over the 180 min digestion, the amount of hydrolysed glucose in the samples increased exponentially before plateauing. Wholemeal flours had somewhat lower glucose curves than other types, suggesting that the pGI associated with them will be somewhat lower.

Apart from Carosella, the examined samples, on average, fell into the low group according to the aforementioned categorization. The low pGI revealed might be attributed to the starch’s amylose concentration observed in the samples (Section 3.4). As a result of their high amylose content, they are less prone to gelatinization resulting in a lower GI [62]. Compared to Risciola and Carosella, soft wheat varieties, the flours obtained from Saragolla durum wheat had a lower pGI, as shown in Table 5. This was consistent with the results found for the amylose/amylopectin ratio (Section 3.4).

Previous works that evaluated the pGI of wheat as raw material have reported higher values (87.3 ± 1.2; 74.4 ± 8.1) than those found in the flours obtained from ancient grains [63,64].

The glycemic response of each food is determined by several factors, such as the ratio of amylose to amylopectin, the degree of processing applied to the food, and the content of fats and proteins in the food [64].

### 3.6. Quality Traits Assessment

**Figure 6:**
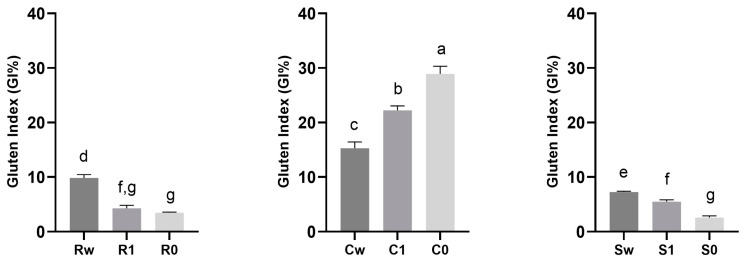
Gluten index (GI%) in flours obtained from ancient grains with different degrees of sifting (whole wheat, type 1, and type 0). Data are expressed as mean ± SD. Different lowercase letters indicate significant differences (*p* < 0.05). Abbreviations: Rw: Risciola whole wheat; R1: Risciola 1; R0: Risciola 0; Cw: Carosella whole wheat; C1: Carosella 1; C0: Carosella 0; Sw: Saragolla whole wheat; S1: Saragolla 1; and S0: Saragolla 0.

The functional properties of wheat mainly depend on the quantity and quality of gluten. Gluten proteins, classified into gliadins (ω, α and γ gliadins), high-molecular-weight (HMW-GS) and low-molecular-weight (LMW-GS) glutenin, are not homogeneously allocated within the seed; indeed, the content of HMW-GS and **γ** gliadin is higher in the middle endosperm, while LMW-GS and other monomeric gliadins are more abundant in the outermost layers of the seed [65]. Consequentially, the different distribution of the functional groups of the gluten could have a different influence on the gluten index and, therefore, on the final quality of the flours, based on variety but also as the result of the different degrees of sifting. Among the three varieties analyzed, Carosella had the highest gluten index, while it is particularly low in the other two varieties with values ranging from 2.60 ± 0.26 to 7.26 ± 0.14 and 3.47 ± 0.11 to 9.84 ± 0.51 for Saragolla and Risciola, respectively, as shown in Figure 6. The gluten index, as the main quality indicator, is usually positively influenced by the proportion of glutenin and the ratio of glutenin to gliadin; nevertheless, the gluten index of the three varieties that are the object of this study, notwithstanding the differences, can be classified as weak with a value of <30% [66].

**Figure 7:**
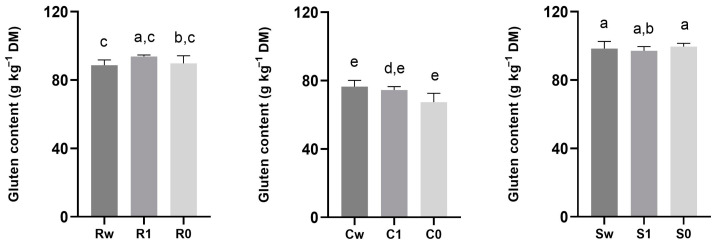
Gluten content (g kg^−^^1^ DM) in flours obtained from ancient grains with different degrees of sifting (whole wheat, type 1, and type 0). Data are expressed as mean ± SD. Different lowercase letters indicate significant differences (*p* < 0.05). Abbreviations: Rw: Risciola whole wheat; R1: Risciola 1; R0: Risciola 0; Cw: Carosella whole wheat; C1: Carosella 1; C0: Carosella 0; Sw: Saragolla whole wheat; S1: Saragolla 1; and S0: Saragolla 0.

The degree of sifting did not significantly affect the gluten content within the same variety; in Saragolla, gluten content ranged from 97.15 ± 2.14 to 99.59 ± 1.64 g kg^−^^1^ DM, while in Risciola, it ranged from 88.64 ± 2.74 to 93.75 ± 0.81 g kg^−^^1^ DM (Figure 7). The lowest gluten content was found in Carosella (67.39 ± 4.51 to 76.49 ± 3.14 g kg^−^^1^ DM; Figure 7).

As reported by Giunta et al., (2020) [67], ancient varieties have a higher and weaker gluten content. This difference can be attributed to genetic-improvement practices in modern varieties that have increased gluten index and protein content [67].

The farinographic test is important to characterize the flour’s behavior during the dough’s mechanical stresses. The parameters that were considered are water absorption, the development time, which represents the time necessary for the dough to reach the optimal consistency; the stability of the flour, which is the time interval in which the dough is kept at the optimal consistency; and the degree of softening expressed in FU, which indicates the tendency of the dough and the gluten to lose consistency (Table 6).

The development time ranges from 1.2 min (C1) to 2.6 min (Sw). For all varieties, regardless of the degree of sifting, the development time is very short. Development time is linked to the time needed for gluten to form adequately (Table 6).

Farinograph stability ranges from 3.1 min (Cw) to 1.6 min (R1) (Table 6). These values indicate low dough stability; higher values of this parameter are associated with more stable doughs, which may thus be employed for long rising times.

The softening degree, associated with the loss of consistency of the dough, is quite high in all the varieties considered, regardless of the degree of sifting, with a minimum and maximum value in Sw and S0 (Table 6).

Water absorption, the water necessary to reach the optimal consistency, increases from type 0 to whole wheat for all varieties considered, ranging from 56. 7% to 59.4 % (Table 6). This parameter is influenced by multiple factors, including gluten and damaged starch. Damaged starch, produced during mechanical milling operations, and gluten increase the amount of water needed to achieve optimal consistency and may be linked to the degree of sifting [68]. A high water absorption is suitable if associated with a low degree of softening.

### 3.7. Microbiological Analysis

Table 7 shows the results for viable microorganisms analyzed in ancient grain flours with different sifting degrees.

Flours from whole ancient grains showed the highest contents of all investigated microbial groups compared to the respective refined flours (type 1 and 0).

Total mesophilic counts recorded the highest values in whole-wheat-flour samples with significantly different values ranging from 4.01 log cfu/g (Cw) to 5.35 log cfu/g (Sw).

These results are in agreement with previous studies claiming that most of the microbial contamination is hosted in the surface of grains, and remains, after milling, more prevalently in the bran-rich fractions [69,70,71]. Presumptive lactobacilli showed microbial load between 2.48 log cfu/g (R0) and 3 log cfu/g (Sw). Our results are similar to those reported by Alfonso et al., (2013) who found viable counts of lactic acid bacteria within wheat flours in the range 2.56–4.64 log cfu/g [70].

Yeasts were only present in the whole-wheat-flour samples with significantly different microbial loads ranging from 1.61 log cfu/g (Rw) to 2.03 (Sw). This microbial group, which, like lactic acid bacteria, plays an important role in the fermentation processes of flours, showed a slightly lower microbial load than that reported by other authors in wheat flours from Australia and Portugal [37,72].

Molds, considered the worst spoilage organisms in flours because they are capable of producing mycotoxins and allergenic spores [73], were found in all flour samples analyzed.

In detail, their values were highest in whole-grain flours, where they ranged from 3.6 cfu/g (Rw) to 4.7 (Sw). Type 0 flours showed lower mold levels not exceeding 3 log cfu/g. Our results are in agreement with Cardoso et al. (2019) who highlighted higher microbial mold values for whole-wheat flour [37]. Presumptive *Bacillus cereus* were also present only in whole-wheat-flour samples with microbial loads ranging between 2.0 and 2.55 log cfu/g flour. The occurrence of *Bacillus* spp. in low-moisture food such as flours and various starchy foods such as potatoes and unhusked rice was found by other authors [74,75]. In addition, Reale et al. (2023) have ascertained a fair biodiversity of *Bacillus* species in cereals from the Mediterranean environment of Southern Italy [19].

*Enterobacteriaceae* were present in samples of whole-wheat and type-1 flours, whereas they were less than 1 log cfu/g in type 0 flours of the different ancient grains.

Total and faecal coliforms were present in all analyzed samples with microbial loads ranging from 1.91 log cfu/g (R0) to 3.95 log cfu/g (Sw) and 1.48 log cfu/g (C0) to 3.19 log cfu/g (Sw), respectively. Coliforms were not detected in any of the flour samples analyzed.

*Salmonella* spp., as in other studies [37,76], was also absent in all flour samples analyzed. As sifting operations proceed, not only the chemical composition but also the microbiological quality of the flours is affected. For all varieties studied, a progressive decrease in microbial load was found from whole-wheat flour to type-1 flour and then to type-0 flour.

Overall, the results of microbiological analyses performed on samples of flour from ancient grains at different degrees of sifting show the safety of the analyzed flours, given that the microbiological parameters were within the legal microbiological criteria set by the European Commission Regulation (EC) No. 2073/2005 [77]. The present study also confirms a decrease in microbial contamination as the outer layers of the grain are removed during the milling process.

## 4. Conclusions

Ancient wheat cultivars have become more popular due to their low input and organic management requirements, as well as their higher nutritional value than modern wheat varieties. Choosing ancient grains also means supporting an artisanal supply chain, buying a local product processed at a lower temperature to preserve the nutrients and ensure quality.

As of right now, there is not enough evidence that ancient wheat flour can partially replace common wheat flour.

In our investigation, the organically grown cereals were crushed with a stone mill using a time/temperature program that avoided overheating of the raw material to safeguard all its constituent parts, yielding wholemeal flours, types 1 and 0.

It was found that all samples investigated had a beneficial nutritional profile, with a greater amylose/amylopectin ratio and a lower glycemic index. The “weak” gluten in all three varieties can be beneficial for the digestive system.

The nutritional and health properties of ancient wheat species will be investigated in vitro, as well as compared to modern wheat varieties.

## Figures and Tables

**Table 1:** Interclass Distance of the three grinding cereals Risciola, Carosella, and Saragolla.

(a)				
Material	Interclass Distance			
	Saragolla	Risciola		Carosella
Risciola	-	-		2.20
Saragolla	-	2.14		3.22
**(b)**				
**Material**	**Recognition Rate (%)**		**Rejection Rate (%)**	
Risciola	100 (7/7)		69 (9/13)	
Carosella	100 (7/7)		76 (10/13)	
Saragolla	100 (6/6)		42 (6/14)	

**Table 2:** Color characteristics, pH, and water activity (a_w_) of ancient grain flours with different degrees of sifting (whole wheat, type 1, and type 0).

Sample	Colorimetric Indices					pH	a_w_
	L*	a*	b*	C*	h*		
**Rw**	67.86 ± 0.36 ^d^	0.54 ± 0.04 ^b^	8.39 ± 0.12 ^f^	8.41 ± 0.13	85.72 ± 0.65 ^i^	6.05 ± 0.04 ^a^	0.467 ± 0.005 ^b^
**R1**	68.91 ± 0.31 ^c^	0.28 ± 0.04 ^c^	8.61 ± 0.03 ^e^	8.61 ± 0.03 ^d^	88.30 ± 0.14 ^g^	6.08 ± 0.01 ^a^	0.476 ± 0.008 ^b^
**R0**	69.04 ± 0.14 ^c^	0.17 ± 0.03 ^d^	9.01 ± 0.08 ^d^	9.01 ± 0.08 ^e^	88.96 ± 0.15 ^f^	6.07 ± 0.02 ^a^	0.486 ± 0.007 ^a,b^
**Cw**	70.51 ± 0.21 ^b^	−0.22 ± 0.01 ^e^	7.82 ± 0.02 ^g^	7.83 ± 0.02 ^b^	91.54 ± 0.05 ^d^	6.07 ± 0.04 ^a^	0.510 ± 0.002 ^a^
**C1**	70.58 ± 0.52 ^b^	−0.27 ± 0.03 ^f^	7.70 ± 0.08 ^h^	7.70 ± 0.08 ^a^	91.96 ± 0.13 ^b^	6.09 ± 0.01 ^a^	0.511 ± 0.011 ^a^
**C0**	71.29 ± 0.16 ^a^	−0.39 ± 0.02 ^g^	7.57 ± 0.09 ^h^	7.57 ± 0.09 ^a^	92.86 ± 0.17 ^a^	6.12 ± 0.05 ^a^	0.513 ± 0.002 ^a^
**Sw**	61.26 ± 0.15 ^g^	0.77 ± 0.12 ^a^	17.30 ± 0.28 ^a^	17.30 ± 0.28 ^h^	87.48 ± 0.42 ^h^	6.10 ± 0.02 ^a^	0.466 ± 0.008 ^b^
**S1**	63.59 ± 0.24 ^f^	0.16 ± 0.05 ^d^	15.72 ± 0.18 ^b^	15.72 ± 0.18 ^g^	89.48 ± 0.18 ^e^	6.11 ± 0.02 ^a^	0.485 ± 0.011 ^a,b^
**S0**	66.18 ± 0.12 ^e^	−0.47 ± 0.01 ^h^	15.00 ± 0.05 ^c^	15.00 ± 0.05 ^f^	91.74± 0.05 ^c^	6.13 ± 0.04 ^a^	0.493 ± 0.006 ^a,b^

Means ± standard deviations of triplicate independent experiments are shown. Within each column, overall means with different superscript letters are significantly different (*p* < 0.05). Abbreviations: Rw: Risciola whole wheat; R1: Risciola 1; R0: Risciola 0; Cw: Carosella whole wheat; C1: Carosella 1; C0: Carosella 0; Sw: Saragolla whole wheat; S1: Saragolla 1; and S0: Saragolla 0.

**Table 3:** Proximate composition of ancient grain flours with different degrees of sifting (whole wheat, type 1, and type 0).

Sample	Moisture (%)	Ash (%)	Lipid (%)	Protein (%)	TDF (%)	SDF (%)	IDF (%)	Carbohydrates (%)
**Rw**	10.62 ± 0.07	2.04 ± 0.05	2.35 ± 0.04 *	10.18 ± 0.01	13.20 ± 0.01 **	1.46 ± 0.04	11.68 ± 0.05 **	61.61 ± 0.10 *
**R1**	11.20 ± 0.08	1.73 ± 0.04	1.46 ± 0.05	10.54 ± 0.03	8.36 ± 0.00	2.16 ± 0.04 *	6.35 ± 0.00 *	66.71 ± 0.11
**R0**	11.46 ± 0.03	1.63 ± 0.02	1.18 ± 0.00	10.38 ± 0.04	8.13 ± 0.02	1.44 ± 0.02	6.85 ± 0.01	67.22 ± 0.06
**Cw**	11.59 ± 0.06 *	1.20 ± 0.08	2.26 ± 0.02 *	8.14 ± 0.05	11.92 ± 0.06 **	1.48 ± 0.03	10.88 ± 0.07 **	64.89 ± 0.13 **
**C1**	12.64 ± 0.00	1.18 ± 0.07	1.88 ± 0.02	8.96 ± 0.05 *	5.98 ± 0.06	1.37 ± 0.03	4.36 ± 0.02 **	69.36 ± 0.11
**C0**	12.71 ± 0.01	1.04 ± 0.00	1.00 ± 0.07	8.06 ± 0.01	6.41 ± 0.02	1.36 ± 0.02	5.05 ± 0.01	70.78 ± 0.07
**Sw**	11.08 ± 0.01	1.96 ± 0.01	2.63 ± 0.01 **	9.58 ± 0.02	10.72 ± 0.01 **	2.64 ± 0.04 *	8.08 ± 0.02 **	64.03 ± 0.03 *
**S1**	11.03 ± 0.05	1.83 ± 0.03	1.61 ± 0.01 *	9.66 ± 0.05	6.14 ± 0.01	2.35 ± 0.03	3.98 ± 0.07 *	69.73 ± 0.08
**S0**	11.13 ± 0.05	1.73 ± 0.03	1.12 ± 0.03	9.71 ± 0.01	5.84 ± 0.02	2.92 ± 0.06	3.07 ± 0.04	70.47 ± 0.07

Data are expressed as mean ± SD. Symbols indicate significance: * *p* < 0.05, and ** *p* < 0.01 vs. each type 0 flour. The absence of symbols indicates non-significant differences between samples. Abbreviations: TDF (Total Dietary Fiber); SDF (Soluble Dietary Fiber); IDF (Insoluble Dietary Fiber); Rw: Risciola whole wheat; R1: Risciola 1; R0: Risciola 0; Cw: Carosella whole wheat; C1: Carosella 1; C0: Carosella 0; Sw: Saragolla whole wheat; S1: Saragolla 1; and S0: Saragolla 0.

**Table 4:** Amylose/amylopectin ratio in samples of flour obtained from ancient grains with different degrees of sifting (whole wheat, type 1, and type 0).

Sample	Amylose/Amylopectin Ratio
**Rw**	0.294 ± 0.009
**R1**	0.286 ± 0.004
**R0**	0.269 ± 0.002
**Cw**	0.265 ± 0.004
**C1**	0.264 ± 0.003
**C0**	0.260 ± 0.000
**Sw**	0.315 ± 0.000
**S1**	0.320 ± 0.002
**S0**	0.301 ± 0.000

Data are expressed as mean ± SD. The absence of symbols indicates non-significant differences between samples. Abbreviations: Rw: Risciola whole wheat; R1: Risciola 1; R0: Risciola 0; Cw: Carosella whole wheat; C1: Carosella 1; C0: Carosella 0; Sw: Saragolla whole wheat; S1: Saragolla 1; and S0: Saragolla 0.

**Table 5:** Predicted glycemic index (pGI) in samples of flour obtained from ancient grains with different degrees of sifting (whole wheat, type 1, and type 0).

Sample	pGI
**Rw**	53.316 ± 0.075
**R1**	53.811 ± 0.124
**R0**	54.900 ± 0.259
**Cw**	53.726 ± 0.110 **
**C1**	53.598 ± 0.086 *
**C0**	59.412 ± 0.026
**Sw**	52.208 ± 0.128
**S1**	52.414 ± 0.079 *
**S0**	53.837 ± 0.001

Data are expressed as mean ± SD of three independent experiments. Symbols indicate significance: * *p* < 0.05, and ** *p* < 0.01 vs. each type 0 flour. The absence of symbols indicates non-significant differences between samples. Abbreviations: Rw: Risciola whole wheat; R1: Risciola 1; R0: Risciola 0; Cw: Carosella whole wheat; C1: Carosella 1; C0: Carosella 0; Sw: Saragolla whole wheat; S1: Saragolla 1; and S0: Saragolla 0.

**Table 6:** Farinographic parameters of flours obtained from ancient grains with different degrees of sifting (whole wheat, type 1, and type 0).

Sample	Water Absorption (%)	Development Time (min)	Farinograph Stability (min)	Farinograph Softening Degree (FU)
**Rw**	66.00 ± 0.20 ^a^	2.10 ± 0.40 ^ab^	2.00 ± 0.00 ^ab^	132.00 ± 1.00 ^c^
**R1**	63.80 ± 0.20 ^b^	1.80 ± 0.00 ^ab^	1.60 ± 0.20 ^b^	138.00 ± 0.00 ^bc^
**R0**	64.90 ± 0.10 ^ab^	1.80 ± 0.20 ^ab^	2.00 ± 0.07 ^b^	141.50 ± 1.50 ^ab^
**Cw**	57.10 ± 0.10 ^d^	2.50 ± 0.20 ^a^	3.10 ± 0.00 ^a^	104.00 ± 1.00 ^d^
**C1**	56.70 ± 0.20 ^d^	1.20 ± 0.00 ^b^	2.80 ± 0.20 ^ab^	107.00 ± 0.00 ^d^
**C0**	54.70 ± 0.20 ^e^	1.50 ± 0.10 ^ab^	2.80 ± 0.10 ^ab^	108.00 ± 1.00 ^d^
**Sw**	59.40 ± 0.40 ^c^	2.60 ± 0.20 ^a^	2.50 ± 0.10 ^ab^	96.00 ± 1.00 ^e^
**S1**	58.60 ± 0.20 ^c^	2.15 ± 0.15 ^ab^	2.50 ± 0.10 ^ab^	108.00 ± 2.00 ^d^
**S0**	56.80 ± 0.40 ^d^	1.70 ± 0.20 ^ab^	1.95 ± 0.05 ^ab^	147.00 ± 1.00 ^a^

Data are expressed as mean ± SD. Different lowercase letters indicate significant differences (*p* < 0.05). Abbreviations: Rw: Risciola whole wheat; R1: Risciola 1; R0: Risciola 0; Cw: Carosella whole wheat; C1: Carosella 1; C0: Carosella 0; Sw: Saragolla whole wheat; S1: Saragolla 1; and S0: Saragolla 0.

**Table 7:** Results of viable microbial counting in flours from ancient grains at different degrees of sifting (whole wheat, type 1, and type 0). Values are expressed as colony-forming units per gram of flour (Log cfu/g).

Sample	Total Mesophilic Count	Presumptive Lactobacilli	*Salmonella* spp.	Yeasts	Molds	Presumptive *B. cereus*	*Enterobacteriaceae*	Fecal Coliforms	Total Coliforms
**Rw**	4.91 ^b^	2.87 ^a,b^	Absent	1.61 ^b^	3.60 ^c^	2.00 ^b^	2.41 ^a^	2.49 ^b^	2.76 ^b^
**R1**	4.58 ^c^	2.65 ^c^	Absent	<1.00	2.70 ^e^	<1.00	1.51 ^c^	1.85 ^d,e^	2.00 ^d^
**R0**	4.06 ^d^	2.48 ^c^	Absent	<1.00	2.30 ^f^	<1.00	<1.00	1.83 ^e^	1.91 ^d^
**Cw**	4.01 ^d^	2.78 ^b^	Absent	1.74 ^b^	3.00 ^d^	2.00 ^b^	2.00 ^b^	1.83 ^e^	2.30 ^c^
**C1**	3.75 ^e^	2.68 ^b,c^	Absent	<1.00	4.00 ^b^	<1.00	1.32 ^d^	1.72 ^e^	2.30 ^c^
**C0**	3.73 ^e^	2.57 ^c^	Absent	<1.00	2.95 ^d^	<1.00	<1.00	1.48 ^f^	1.97 ^d^
**Sw**	5.35 ^a^	3.00 ^a^	Absent	2.03 ^a^	4.70 ^a^	2.55 ^a^	2.99 ^a^	3.19 ^a^	3.95 ^a^
**S1**	5.03 ^b^	2.92 ^a^	Absent	<1.00	4.08 ^b^	<1.00	1.65 ^c^	2.00 ^c^	2.00 ^d^
**S0**	4.90 ^b^	2.75 ^b^	Absent	<1.00	3.00 ^d^	<1.00	<1.00	1.85 ^d,e^	2.00 ^d^

Within all columns, for each microbial group, means followed by different letters are significantly different (*p* < 0.05). Abbreviations: Rw: Risciola whole wheat; R1: Risciola 1; R0: Risciola 0; Cw: Carosella whole wheat; C1: Carosella 1; C0: Carosella 0; Sw: Saragolla whole wheat; S1: Saragolla 1; and S0: Saragolla 0.

## Data Availability

The data used to support the findings of this study can be made available by the corresponding author upon request.
